# Increased serum catalytic iron may mediate tissue injury and death in patients with COVID-19

**DOI:** 10.1038/s41598-021-99142-x

**Published:** 2021-10-04

**Authors:** Vipul Chakurkar, Mohan Rajapurkar, Suhas Lele, Banibrata Mukhopadhyay, Valentine Lobo, Ramakrishna Injarapu, Muddassir Sheikh, Bharatkumar Dholu, Arpita Ghosh, Vivekanand Jha

**Affiliations:** 1 Renal Unit, Department of Medicine, KEM Hospital, Sardar Moodliar Road, Rasta Peth, Pune, Maharashtra 411011 India; 2 Muljibhai Patel Society for Research in Nephro-Urology, Nadiad, India; 3 Department of Medicine, KEM Hospital, Pune, India; 4 The George Institute for Global Health, India, UNSW, New Delhi, India; 5 Manipal Academy of Higher Education, Manipal, India; 6 School of Public Health, Imperial College, London, UK

**Keywords:** Infectious diseases, Prognostic markers, Molecular biology, Biomarkers, Molecular medicine, Pathogenesis

## Abstract

The pathophysiology and the factors determining disease severity in COVID-19 are not yet clear, with current data indicating a possible role of altered iron metabolism. Previous studies of iron parameters in COVID-19 are cross-sectional and have not studied catalytic iron, the biologically most active form of iron. The study was done to determine the role of catalytic iron in the adverse outcomes in COVID-19. We enrolled adult patients hospitalized with a clinical diagnosis of COVID-19 and measured serum iron, transferrin saturation, ferritin, hepcidin and serum catalytic iron daily. Primary outcome was a composite of in-hospital mortality, need for mechanical ventilation, and kidney replacement therapy. Associations between longitudinal iron parameter measurements and time-to-event outcomes were examined using a joint model. We enrolled 120 patients (70 males) with median age 50 years. The primary composite outcome was observed in 25 (20.8%) patients—mechanical ventilation was needed in 21 (17.5%) patients and in-hospital mortality occurred in 21 (17.5%) patients. Baseline levels of ferritin and hepcidin were significantly associated with the primary composite outcome. The joint model analysis showed that ferritin levels were significantly associated with primary composite outcome [HR (95% CI) = 2.63 (1.62, 4.24) after adjusting for age and gender]. Both ferritin and serum catalytic iron levels were positively associated with in-hospital mortality [HR (95% CI) = 3.22 (2.05, 5.07) and 1.73 (1.21, 2.47), respectively], after adjusting for age and gender. The study shows an association of ferritin and catalytic iron with adverse outcomes in COVID-19. This suggests new pathophysiologic pathways in this disease, also raising the possibility of considering iron chelation therapy.

## Introduction

As of writing, a total of 241 million individuals have been infected with the novel beta-coronavirus SARS-CoV-2 worldwide, with over 4 million deaths^1^. COVID-19 (coronavirus disease 2019) has varied clinical manifestations, ranging from asymptomatic infection to severe acute respiratory failure, multi-organ dysfunction, and death. The mortality is high amongst patients with severe disease, especially those who develop respiratory failure or acute kidney injury (AKI)^2^. The pathophysiology and the factors that determine the disease severity are not yet clear, with the main pathways postulated to be responsible for tissue injury being the direct cytopathic effect of the virus, immune dysregulation leading to cytokine storm, endothelial cell damage, and thrombo-inflammation^3^.

Non-transferrin or non-ferritin bound iron, also called catalytic iron, leads to the generation of reactive oxygen species such as hydroxyl radicals through the Fenton reaction^4^. High catalytic iron levels in plasma are associated with mortality and adverse clinical events in patients with a variety of acute illnesses, including acute coronary syndromes^5,6^, cardiogenic shock^7^, and multi-organ failure with AKI^8,9^.

Previous cross-sectional studies of iron parameters in COVID-19 have reported an association between one-time measurements of these parameters with the poor outcomes^10–16^. Severe COVID-19 is also thought to be one of the hyperferritinemic syndromes. There is evidence showing an association between high levels of hepcidin and ferritin and the severe forms of this disease^10–12^. Nai et al. in a study of 111 Italian patients with COVID-19, found high hepcidin levels to be associated with death^11^. In another study of 50 patients, Zhou et al. showed that patients with severe COVID-19 had higher levels of hepcidin and ferritin^12^.

We hypothesized that serum catalytic iron (SCI) levels increase in COVID-19 and are associated with increased disease severity and adverse outcomes, including death.

## Results

**Figure 1:**
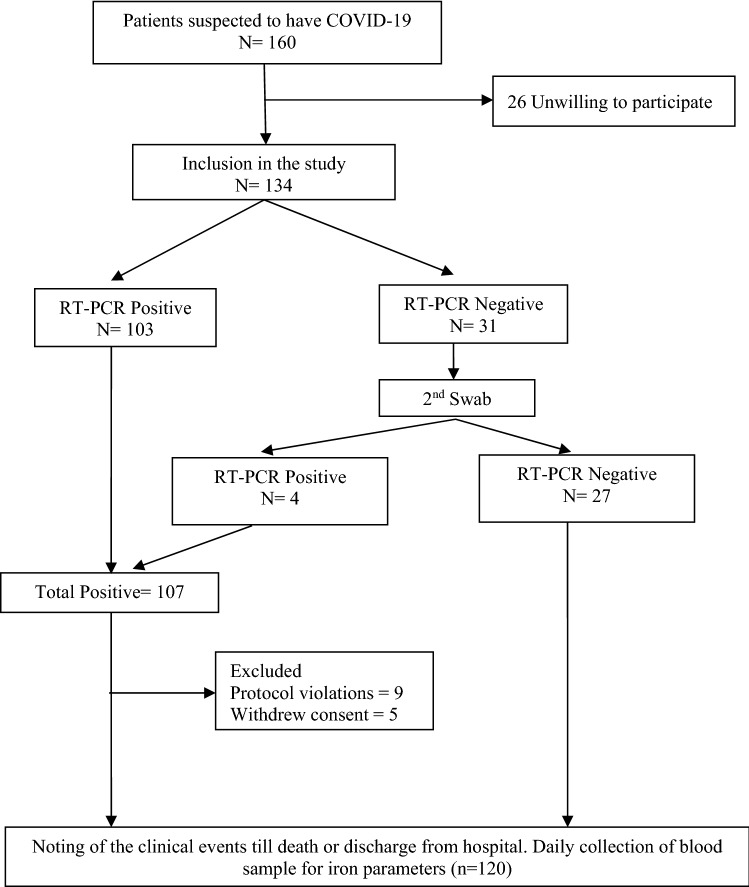
Shows the flow of the study.

Of the total of 160 patients admitted with SARS-CoV2 infection at King Edward Memorial Hospital, Pune, 134 consented. The final analysis included 120 participants (Fig. 1). The baseline characteristics of the participants are presented in Table 1. Their median age was 50 years (interquartile range (IQR) = 35.8, 64 years), 70 (58.3%) of them were males, 35 (29.2%) were diabetic, and 41 (34.2%) were hypertensive. Patients presented to the hospital at a median of 4 days (IQR = 3, 5 days) after symptom onset. Of the 120 patients, 93 (77.5%) were positive by SARS-CoV-2 real-time polymerase chain reaction (RT-PCR), and the remaining 27 (22.5%) were clinically suspected of having COVID-19 on the basis of fever, acute severe respiratory illness and an abnormal chest roentgenogram.

**Table 1:** Baseline characteristics of study participants, by study outcomes.

Characteristics	Entire cohort (n = 120)	Patients having primary composite outcome* (n = 25)	Non-survivors (N = 21)
Age	50.0 (35.8, 64.0)	60.0 (44.0, 72.0)	60.0 (51.0, 74.0)
Male sex, n (%)	70 (58.3)	16 (64)	13 (61.9)
Diabetes, n (%)	35 (29.2)	6 (24)	4 (19)
Hypertension, n (%)	41 (34.2)	12 (48)	11 (52.4)
Ischemic heart disease, n (%)	11 (9.2)	3 (12)	2 (9.5)
Lung disease, n (%)	7 (5.8)	1 (4)	1 (4.7)
RT-PCR positive, n (%)	93 (77.5)	19 (76)	17 (81)
Days since symptom onset	4.0 (3.0, 5.0)	4.0 (4.0, 5.0)	4.0 (4.0, 5.0)
N:L Ratio	3.2 (2.2, 6.0)	6.2 (3.1, 13.3)	6.2 (3.1, 11.7)
CRP (mg/L)	57.7 (20.0, 142.0)	153.0 (84.5, 160.0)	148.0 (85.2, 160.0)
IL-6 (pg/ml)	59.0 (36.5, 135.0)	110.0 (57.0, 206.8)	112.5 (59.8, 242.2)

*Primary composite outcome is a composite of in-hospital mortality, need for mechanical ventilation and need for kidney replacement therapy.

Values are expressed as median (interquartile range) or count (percentage).

The values of CRP, N: L ratio, IL-6 and iron parameters mentioned here are baseline measurements.

CRP, N: L ratio and IL-6 are missing for 11, 3 and 36 participants.

CRP-C-Reactive Protein; IL-6- interleukin-6; N:L ratio- neutrophil-to-lymphocyte ratio; RT-PCR-real time polymerase chain reaction.

### Outcomes

Over the course of hospital stay, 70 (58.3%) patients required oxygen therapy; 40 (33.3%) needed ICU care with a median length of ICU stay of 8.5 days (IQR = 5, 14 days). The median length of stay in hospital was 9 days (IQR = 6, 11.25 days). The primary composite outcome was observed in 25 (20.8%) patients, 21 patients (17.5%) died, 21 (17.5%) needed mechanical ventilation, 12 (10%) developed AKI and 7 (5.8%) required kidney replacement therapy (KRT). The baseline characteristics of the patients who met with the primary composite outcome and death are described in Table 1.

### Iron parameters

Participants who met with the primary composite outcome had lower baseline levels of serum iron (SI) [23.3 (15.5, 40.5) vs. 31.9 (20.0, 61.7), *p* = 0.03] and total iron-binding capacity (TIBC) [225.1 (191.0, 254.5) vs. 268.2 (219.6, 322.2), *p* < 0.01] and higher baseline levels of ferritin [720.6 (251.6, 1406.0) vs. 260.3 (94.3, 507.9), *p* < 0.01] and hepcidin [221.6 (150.6, 315.3) vs. 92.2 (36.0, 232.2), *p* < 0.01], as compared to those who had not met with the outcome (Table 2). Comparison of those who died with the survivors revealed higher ferritin and hepcidin levels and lower serum iron and TIBC levels at baseline. No difference in baseline levels of SCI was detected between the groups who met with the outcome and those who did not (Table 2).

**Table 2:** Distribution of iron parameters, by outcome status.

Iron parameter	Primary composite outcome*			Death		
	No (n = 95)	Yes (n = 25)	*P*-value	No (n = 99)	Yes (n = 21)	*P*-value
SI (μg/dl)	31.9 (20.0, 61.7)	23.3 (15.5, 40.5)	0.03	31.9 (19.7, 61.7)	23.3 (18.4, 29.9)	0.04
TIBC (μg/dl)	268.2 (219.6, 322.2)	225.1 (191.0, 254.5)	< 0.01	267.7 (220.9, 320.4)	216.5 (191.0, 254.5)	< 0.01
TSAT (%)	12.7 (8.2, 24.3)	9.9 (7.8, 20.1)	0.31	12.7 (7.8, 24.3)	9.9 (8.6, 20.1)	0.41
Ferritin (ng/ml)	260.3 (94.3, 507.9)	720.6 (251.6, 1,406.0)	< 0.01	251.6 (103.0, 507.9)	725.7 (280.5, 1,406.0)	< 0.01
Hepcidin (ng/ml)	92.2 (36.0, 232.2)	221.6 (150.6, 315.3)	< 0.01	105.8 (37.3, 232.2)	225.1 (163.4, 315.3)	< 0.01
SCI (μmol/L)	0.39 (0.3, 0.64)	0.4 (0.34, 0.66)	0.35	0.39 (0.3, 0.64)	0.43 (0.36, 0.67)	0.24

*Primary composite outcome is a composite of in-hospital mortality, need for mechanical ventilation and need for kidney replacement therapy.

Values are expressed as median (interquartile range).

SI- serum iron; SCI- serum catalytic iron; TIBC- total iron binding capacity, TSAT- transferrin saturation.

### Severity of the disease

A total of 22 patients had mild disease, whereas 57 and 41 patients were classified into moderate and severe categories, respectively. The group characteristics are described in Table 3. Patients with severe disease were older and included more hypertensives compared to those with mild disease. Baseline CRP and neutrophil-to-lymphocyte ratio were associated with disease severity. RBC counts did not differ between the groups, and no excess bleeding was observed in the severe group. The levels of ferritin, hepcidin, and TIBC differed significantly across the groups while those of SI, TSAT, and SCI did not (Table 3).

**Table 3:** Baseline characteristics of study participants according to disease severity (per the highest WHO Score).

	Severity			
	Mild (n = 22)	Moderate (n = 57)	Severe (n = 41)	*P*-value
Age	36.0 (30.2, 48.8)	50.0 (36.0, 61.0)	59.0 (48.0, 70.0)	< 0.01
Male sex	14 (63.6)	30 (52.6)	26 (63.4)	0.48
Diabetes	3 (13.6)	21 (36.8)	11 (26.8)	0.12
Hypertension	2 (9.1)	19 (33.3)	20 (48.8)	< 0.01
RT-PCR positive	22 (100.0)	40 (70.2)	31 (75.6)	< 0.01
Days since symptom onset	2.0 (1.2, 3.0)	4.0 (3.0, 5.0)	4.0 (3.0, 5.0)	< 0.01
N:L Ratio	2.3 (1.5, 2.6)	3.2 (2.2, 6.1)	4.9 (3.0, 11.0)	< 0.01
RBC Count	4.70 (0.74)	4.59 (0.72)	4.50 (0.77)	0.63
CRP (mg/L)	16.8 (3.5, 48.1)	49.0 (11.2, 125.0)	136.5 (55.7, 160.0)	< 0.01
IL-6 (pg/ml)	65.0 (43.8, 201.2)	47.5 (24.5, 116.2)	84.0 (37.8, 145.0)	0.22
SI (μg/dl)	37.0 (27.0, 62.8)	28.6 (17.6, 60.0)	28.7 (18.8, 43.9)	0.19
TIBC (μg/dl)	294.6 (267.9, 349.5)	261.9 (213.3, 322.7)	222.9 (190.1, 265.7)	< 0.01
TSAT (%)	11.3 (9.2, 22.9)	11.9 (7.2, 22.7)	12.7 (8.9, 22.9)	0.73
Ferritin (ng/ml)	206.9 (27.2, 281.1)	249.9 (88.0, 524.1)	537.5 (260.3, 946.5)	< 0.01
Hepcidin (ng/ml)	51.4 (25.1, 148.9)	106.5 (38.4, 240.5)	208.1 (111.5, 302.5)	< 0.01
SCI (μmol/L)	0.33 (0.3, 0.39)	0.39 (0.29, 0.59)	0.43 (0.36, 0.73)	0.058

Continuous data were compared using Kruskal–Wallis ANOVA and categorical data were compared using chi-square test or Fisher’s exact test.

Summaries are expressed as median (interquartile range) or counts (percentage).

The values of CRP, N:L ratio and iron parameters mentioned here are done at baseline.

Mild disease: Highest WHO score 0–3; moderate disease: 4 & 5; Severe: 6 or above.

CRP- C-Reactive Protein; IL-6- interleukin-6; N:L ratio- neutrophil-to-lymphocyte ratio; RT-PCR- real time polymerase chain reaction; SI- serum iron; SCI- serum catalytic iron; TIBC- total iron binding capacity, TSAT- transferrin saturation.

### Association of baseline iron parameters with time-to-event outcomes

In an unadjusted analysis, baseline ferritin and hepcidin were associated with the composite primary outcome and in-hospital mortality [(hazard ratio- HR (95% confidence interval CI) for log-transformed ferritin and hepcidin = 1.94 (1.34, 2.80) and 1.84 (1.19, 2.85), respectively for the primary composite outcome and 2.21 (1.36, 3.59) and 1.92 (1.11, 3.33), respectively for in-hospital mortality]. After adjustment for age and gender, both the parameters retained their association with the outcomes [adjusted HR (95% CI) = 1.85 (1.24, 2.74) and 1.64 (1.03, 2.60), respectively for the primary composite outcome and 2.18 (1.31, 3.61) and 1.75 (1.01, 3.06), respectively for in-hospital mortality]. Additionally, after adjusting for age and gender, baseline SCI levels were associated with the primary composite outcome and in-hospital mortality [adjusted HR for log-transformed SCI = 1.36 (1.00, 1.85) for primary composite outcome and 1.68 (1.17, 2.42) for in-hospital mortality] (Table 4). Mean and maximum iron parameters measured over the study period are described in Table E1.

**Table 4:** Hazard ratios for baseline iron parameters from unadjusted and adjusted Cox proportional hazard models for primary and secondary outcomes.

Iron parameter^†^	Unadjusted model		Adjusted^¥^ model	
	Primary composite outcome*	Death	Primary composite outcome*	Death
SI	0.60 (0.32, 1.12)	0.51 (0.23, 1.12)	0.61 (0.33, 1.14)	0.52 (0.23, 1.16)
TIBC	0.34 (0.13, 0.87)	0.20 (0.06, 0.67)	0.42 (0.14, 1.26)	0.24 (0.06 0.93)
TSAT	0.85 (0.47, 1.53)	0.82 (0.40, 1.70)	0.74 (0.39, 1.40)	0.73 (0.33, 1.60)
Ferritin	1.94 (1.34, 2.80)	2.21 (1.36, 3.59)	1.85 (1.24, 2.74)	2.18 (1.31, 3.61)
Hepcidin	1.84 (1.19, 2.85)	1.92 (1.11, 3.33)	1.64 (1.03, 2.60)	1.75 (1.01, 3.06)
SCI	1.26 (0.95, 1.66)	1.43 (1.05, 1.96)	1.36 (1.00, 1.85)	1.68 (1.17, 2.42)

*Primary composite outcome is a composite of in-hospital mortality, need for mechanical ventilation and need for kidney replacement therapy.

^¥^adjusted for age and gender.

^†^ log-transformed.

SI- serum iron; SCI- serum catalytic iron; TIBC- total iron binding capacity, TSAT- transferrin saturation.

### Longitudinal iron measurements

**Figure 2:**
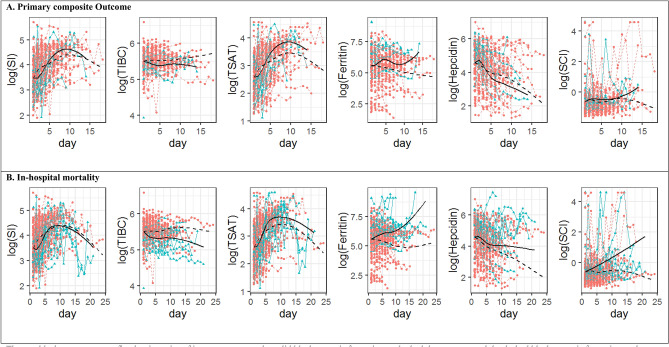
Trajectories of iron parameters, by outcome status. (**a**) The two black curves present fitted trajectories of iron parameters—the solid black curve is for patients who had the event and the dashed black curve is for patients who recovered. (**b**) The blue triangles and lines present iron measurements and trajectories of individual patients who had the outcome. The pink dots and lines present iron measurements and trajectories of individual patients who did not have the outcome. (**c**) Notable is the finding that levels of ferritin and SCI continue to rise in patients who died while the survivors did not have the rise in levels. SCI–serum catalytic iron, SI–serum iron, TIBC- total iron binding capacity, TSAT–transferrin saturation.

Trajectories of natural logarithms of iron parameters were plotted from the day of admission. Fitted trajectories were plotted for patients with or without primary composite outcome and also for survivors and non-survivors. It was noted that patients who died had an increasing trend of SCI & ferritin levels while patients who recovered did not experience this rising trend (Fig. 2).

### Joint modeling

In order to assess the effect of longitudinal measurements of iron parameters on time-to-event outcomes, we used joint modeling. The association is presented as HR (95% CI) adjusted for age and gender in Table 5. Ferritin was positively associated with primary composite outcome: a one-unit increase in ferritin on the log scale resulted in a 2.sixfold increased hazard of primary composite outcome (95% CI 1.62, 4.24). Both log ferritin [adjusted HR (95% CI) = 3.22 (2.05, 5.07)] and log SCI levels [1.73 (1.21, 2.47)] had a significant association with mortality as well. Findings were similar when we adjusted for additional covariates at baseline—medical history of diabetes, hypertension, heart disease, lung disease, day of presentation after symptom onset and RT-PCR status (Table E2).

**Table 5:** Hazard ratios (95% confidence interval) from joint model of longitudinal iron measurements (log-transformed) and time-to-event data, adjusted for age and gender.

Iron Parameter^†^	Primary composite outcome*	Death
SI	0.43 (0.17, 1.11)	0.77 (0.35, 1.71)
TIBC	0.22 (0.06, 0.79)	0.21 (0.05 0.95)
TSAT	0.55 (0.22, 1.38)	1.04 (0.37, 2.92)
Ferritin	2.63 (1.62, 4.24)	3.22 (2.05, 5.07)
Hepcidin	1.40 (0.87, 2.26)	1.38 (0.94, 2.03)
SCI	1.35 (0.90, 2.04)	1.73 (1.21, 2.47)

*Primary composite outcome is a composite of in-hospital mortality, need for mechanical ventilation and need for kidney replacement therapy.

^†^ log-transformed.

SI- serum iron; SCI- serum catalytic iron; TIBC- total iron binding capacity, TSAT- transferrin saturation.

### Sensitivity analysis for very high SCI levels

We found several SCI values to be much higher compared to our previous experience in various critically ill populations. In the past, we have not observed values of more than 10 μmol/L; hence a possibility of inadvertent iron contamination could not be reasonably ruled out. Values greater than 10 μmol/L were seen in 13 patients, of which 7 had died. Hence joint model with sensitivity analysis was done—we refitted the joint model after excluding SCI values > 10 μmol/L. In this analysis, the HR for primary outcome was not statistically significant [HR (95% CI) = 1.72 (0.67, 4.42)] while that for mortality was significant [HR (95% CI) = 2.64 (1.13, 6.14)].

## Discussion

In this prospective longitudinal comprehensive analysis of multiple iron parameters in patients with COVID-19, we show that several markers of iron metabolism are associated with adverse outcomes. Baseline levels of ferritin and hepcidin were associated with adverse outcomes, including death, need for mechanical ventilation and AKI. The association between trajectories of SCI and ferritin with adverse outcomes, including death in this cohort of patients, is a novel finding.

Iron is the most abundant transitional metal in the human body. Most of it is bound to macromolecules like hemoglobin, ferritin, transferrin, and other iron-containing proteins. A tiny amount, existing in unbound form and termed labile or catalytic iron, was originally considered as a transitional pool of extracellular and cellular iron ^8^. Through Fenton and Haber–Weiss reactions, catalytic iron combines with superoxide to form the reactive hydroxyl radical and damage lipids, proteins, and DNA.

Iron is also required for viral replication. Iron overload is associated with poorer prognosis in patients with hepatitis B, C, and human immunodeficiency virus infections^18^. It is suggested that SARS-CoV-2 might require iron for replication and other functions^19^, providing a potential mechanism for greater pathogenicity in the presence of high SCI. Our findings support the role of ferrotoxicity in COVID-19 and the notion that SCI is an indicator of poor outcomes in COVID-19. Our study did not identify the source of SCI. Elevated levels can result from tissue injury either from a direct cytopathic effect or as a result of inflammation or ischemia leading to a sudden release of intracellular stores of iron.

The elevation in ferritin was identified early in the course of the pandemic. Association of high ferritin levels with severity of the illness and mortality^2,10,20–22^ led to the practice of routine measurement of serum ferritin in COVID-19. However, the reason for the early elevation of ferritin is not known^23^. Ferritin can be an acute phase reactant. It could also act as a source of labile iron in the presence of superoxide produced during the hyperinflammatory state, or act as a protective molecule that can mitigate ferrotoxicity by binding free iron. Similarly, hepcidin production is upregulated in iron-overload states to block iron absorption from the gut and macrophage iron recycling as a protective mechanism. The absence of this elevation of hepcidin in the present study might indicate a failure of this protective mechanism.

Despite several clinical trials, effective treatment options for COVID-19 are few, with the only treatment with high-quality evidence being the use of dexamethasone^24^. While a number of therapeutic options are being explored to blunt the immunological hyperactivity, the possibility of blunting the disease severity using iron chelation therapy is worth considering^19^. Iron chelation is shown to be effective in blunting the in-vitro production of IL-6 following infection with influenza virus and *Chlamydia pneumonia*. It can reduce mortality in murine models of septic shock^25^. Our study provides a rationale for considering iron chelation therapy in COVID-19.

The main strength of our study is the longitudinal measurement of a comprehensive set of iron parameters. Joint models are an improvement over traditional models because they consider the longitudinal observations of covariates that might predict of the event of interest. Thus, predictions from joint models have greater accuracy^26^. The joint model analysis was able to confirm the association between SCI levels and adverse outcomes, including mortality. Similarly, there was an association of ferritin levels with the same outcomes.

There are a few limitations, such as a relatively small sample size, and a smaller number of events, especially the need for KRT. We included patients diagnosed with COVID-19 according to the prevailing public health standards, which included RT-PCR negative patients who had similar clinical features and characteristic imaging findings. Such clinically suspected but RT-PCR negative population was included in other large COVID-19 clinical trials. Moreover, there was no change in the hazard ratios when we performed joint modeling of longitudinal iron measurements and time-to-event data, adjusted for RT-PCR test result along with other parameters (Table E3). We did not measure other relevant iron markers such as plasma haptoglobin and hemopexin. Finally, although in the multivariate model we adjusted for several possible confounders, the possibility of residual confounding from unmeasured variables cannot be ruled out.

In conclusion, we show the association of several markers of iron metabolism with adverse outcomes including death, need for mechanical ventilation, and AKI in COVID-19. In particular, the association of the trajectories of SCI and ferritin with poor outcomes may potentially play a role in the pathophysiologic pathway, raising the possibility of considering iron chelation therapy.

## Methods

This prospective study was conducted at King Edward Memorial Hospital, Pune, India, between, June 24 and August 7, 2020. The study protocol was approved by the Ethics Committee of KEM Hospital Research Center, Pune, and was registered with Clinical Trial Registry of India (registration no. CTRI/2020/08/027,419) and conducted as per Declaration of Helsinki. All adult (> 18 years of age) patients admitted with SARS-CoV2 infection (either confirmed by RT-PCR or clinically suspected on the basis of fever, acute severe respiratory illness and abnormal chest roentgenogram) were invited to participate. Those who provided a written informed consent were enrolled in the study. Patients known to have pre-existing chronic kidney disease stage 3 and above were excluded.

As per the prevalent regulations, all patients, including asymptomatic individuals were admitted and were treated according to the local protocols. We divided disease severity according to the highest WHO score during the hospital stay (maximum score 1 to 3 as mild, 4 and 5 as moderate, and 6 or more as severe)^17^. The decision to admit to the critical care unit, provide mechanical ventilation, and initiating kidney replacement therapy (KRT) were at the discretion of the treating teams. All patients were followed up till they were discharged or died. Clinical events (such as the need for oxygen therapy, non-invasive ventilation, mechanical ventilation, need for dialysis support, or vasopressor use) were recorded daily, and WHO class was determined each day. AKI was defined as per Kidney Disease Improving Global Outcomes (KDIGO) criteria^27^. Patients were discharged from the hospital if they were afebrile and did not require oxygen therapy for 3 consecutive days. Starting from the day of admission, 3 ml blood was collected daily in a plain vacutainer from all patients, allowed to clot, and centrifuged. Separated serum was transported to the analytical laboratory at Muljibhai Patel Society for Research in Nephro-Urology, Nadiad, Gujarat, India, and stored at -70^0^C till analysis. All samples were labeled with a unique code and thawed in batches for analysis. The laboratory team was not informed about the clinical condition of the patients or their outcomes.

The primary outcome was a composite of in-hospital mortality, need for mechanical ventilation, and need for KRT. Secondary outcomes were individual components of the primary outcome (death, need for mechanical ventilation, KRT), and the development of severe disease (WHO score 6 or more).

### Laboratory methods used for measuring iron parameters

Bleomycin detectable iron assay was used to measure SCI levels (expressed in µmoles/liter) as previously reported from our laboratory^28^. All reagents except bleomycin were treated overnight with Chelex100 (Bio-Rad) resin to remove possible iron contamination. This method is sensitive and specific to measure catalytic iron in biological fluids such as serum, and does not detect protein-bound iron^29^.

Fully automated clinical chemistry analyzer AU 480, Beckman, USA was used to analyze total SI (micrograms/deciliter) [OSR 6286] and unsaturated iron-binding capacity (UIBC) [OSR 61,205, from Beckman Coulter, USA]. Total iron-binding capacity (TIBC, micrograms/deciliter) was calculated as a sum of SI and UIBC. Transferrin saturation (TSAT, in %) was calculated by dividing SI by TIBC.

We used an automated CLIA platform (Maglumi 2000, SNIBE, Ref: 130201001 M) to measure the serum ferritin level (in nanograms/milliliters). Serum hepcidin levels (in nanograms/milliliters) were measured using monoclonal ELISA (Intrinsic Life Sciences, La Jola, USA). The coefficients of variation and lower limits of detection of above mentioned assays are mentioned in Table E4.

### Statistical analysis

We described the sociodemographic, clinical, and laboratory characteristics using descriptive data [Categorical variables as counts and percentages; continuous data as either mean and standard deviation or median and inter-quartile range (IQR)]. Participants were categorized as having a mild, moderate, and severe disease based on WHO scores^17^. Baseline characteristics were compared using chi-square and Kruskal–Wallis tests. The distributions of iron parameters at baseline in participants with and without the primary composite were compared using the Mann–Whitney U test. We used Cox proportional hazards model to assess the association between baseline iron parameters and time to primary composite outcome and death. The longitudinal data on iron parameters were described using median and IQR of summary measures—mean, median, minimum, and maximum over repeated measurements. We also examined the association between these summary measures of iron parameters and the time-to-event outcomes. Graphs showing individual and fitted trajectories for participants with and without the outcome are presented.

To examine the association between the longitudinal biomarkers and the time-to-event outcomes, we used joint analysis. We used log-transformed iron parameter values as outcomes in the linear mixed-effects model. Both models were adjusted for age and gender and additional covariates such as comorbidities and day of presentation after symptom onset in a sensitivity analysis. To examine the sensitivity of the findings to high SCI values, the joint models for SCI was refitted after excluding SCI values > 10 μmol/L.

## Data sharing

Statistical source code used to generate estimates can be obtained from Dr. Arpita Ghosh, aghosh@georgeinstitute.org.in.

## Supplementary Information

Supplementary Information.
