# Supplementary material for: Increased serum catalytic iron may mediate tissue injury and death in patients with COVID-19
Source: Sci Rep. 2021 Oct 4;11:19618. doi: 10.1038/s41598-021-99142-x (PMC8490366; doi:10.1038/s41598-021-99142-x)
Supplement: Supplementary file 1 — Supplementary Information. [file 41598_2021_99142_MOESM1_ESM.docx]

**Table E1: Median (IQR) of summaries over time – mean and maximum, of iron measurements.**

|  | **SI**  **(μg/dl)** | **TIBC**  **(μg/dl)** | **TSAT (%)** | **Ferritin**  **(ng/ml)** | **SCI**  **(μmol/L)** | **Hepcidin**  **(ng/ml)** |
| --- | --- | --- | --- | --- | --- | --- |
| **All patients** | | | | | | |
| **Mean** | 56.82  (33.64, 81.33) | 252.49  (205.46, 305.43) | 23.7  (11.91, 32.95) | 302.05  (131.32, 634.35) | 0.48  (0.33, 0.79) | 98.02  (36.09, 172.67) |
| **Maximum** | 86.69  (43.16, 124.84) | 280.14  (233.62, 323.41) | 33.72  (15.97, 51.48) | 404.16  (207.06, 1030.9) | 0.69  (0.37, 1.64) | 153.34  (59.91, 301.14) |
| **Patients having primary composite outcome** | | | | | | |
| **Mean** | 40.83  (29.81, 70.42) | 201.75  (163.39, 240.4) | 22.57  (17.13, 30.29) | 745.4  (370.43, 1641.6) | 0.74  (0.46, 2.66) | 167.83  (120.32, 315.34) |
| **Maximum** | 88.87  (34.69, 166.75) | 234.57  (206.19, 274.25) | 52.05  (20.08, 68.55) | 1421.9  (733.2, 3779) | 1.77  (0.52, 6.79) | 302.45  (210.23, 504.89) |
| **Non-survivors** | | | | | | |
| **Mean** | 43.77  (30.91, 70.42) | 196.12  (155.75, 240.4) | 25.04  (18.94, 30.41) | 892.83  (493.22, 2759.98) | 0.84  (0.52, 3.76) | 192.94  (123.05, 315.34) |
| **Maximum** | 96.39  (43.87, 166.75) | 234.57  (191.02, 274.25) | 53.15  (28.43, 72.05) | 1553.3  (1007.9, 8605) | 2.02  (0.9, 31.8) | 302.45  (221.6, 504.89) |

IQR- interquartile range, SCI- serum catalytic iron, SI- serum iron, TIBC- total iron binding capacity, TSAT- transferrin saturation.

**Table E2: Hazard ratios (with 95% confidence interval) from joint model of longitudinal iron measurements and time-to-event data, adjusted for age, gender, RT-PCR test result, medical history of diabetes, hypertension, heart disease, lung disease, and day of presentation after symptom onset.**

| **Iron parameter*** | **Primary composite outcome** | **Death** |
| --- | --- | --- |
| **SI** | 0.42 (0.14, 1.24) | 0.95 (0.39, 2.33) |
| **TIBC** | 0.34 (0.07, 1.66) | 0.27 (0.06, 1.28) |
| **TSAT** | 0.46 (0.15, 1.38) | 1.02 (0.32, 3.25) |
| **Ferritin** | 2.71 (1.51, 4.84) | 3.12 (1.95, 4.98) |
| **Hepcidin** | 1.37 (0.83, 2.26) | 1.54 (0.97, 2.44) |
| **SCI** | 1.24 (0.79, 1.96) | 1.98 (1.35, 2.91) |

^*^Iron parameters are log-transformed

RT-PCR- real-time polymerase chain reaction;SI- serum iron; SCI- serum catalytic iron; TIBC- total iron binding capacity, TSAT- transferrin saturation.

**Table E3: comparison of RT-PCR positive and negative patients.**

|  | **RT-PCR positive (n=93)** | **RT-PCR negative (n=27)** | **p value** |
| --- | --- | --- | --- |
| **Age (years)** | 49 (35, 61) | 56 (42.5, 69.5) | 0.17 |
| **Male gender, n (%)** | 54 (58.1) | 16 (59.3) | 0.91 |
| **DM, n (%)** | 23 (24.8) | 12 (44.4) | 0.04 |
| **HT, n (%)** | 31 (33.3) | 10 (37) | 0.72 |
| **Day of presentation (after symptom onset)** | 4 (3, 5) | 3 (3, 4) | 0.32 |
| **Abnormal chest X-ray, n (%)** | 59 (63.4) | 14 (51.8) | 0.27 |
| **Hypoxia on presentation, n (%)** | 48 (51.6) | 11 (40.7) | 0.31 |
| **Baseline N:L Ratio** | 2.78 (2.17, 5.45) | 4.63 (3.20, 11.04) | 0.03 |
| **Baseline CRP (mg/L)** | 57.7 (21.2, 135.6) | 64.5 (9.23, 158) | 0.97 |
| **Baseline IL-6 (pg/ml)** | 55 (37, 128) | 95 (42.5, 430) | 0.20 |
| **Baseline SI (μg/dl)** | 28.21 (18.84, 49.95) | 42.42 (22.36, 65.74) | 0.08 |
| **Baseline TIBC (μg/dl)** | 263.6 (218.55, 296.91) | 225.14 (196.71, 324.97) | 0.54 |
| **Baseline TSAT (%)** | 11.44 (7.38, 21.81) | 17.39 (9.28, 29.68) | 0.08 |
| **Baseline Ferritin (ng/ml)** | 279.6 (166.1, 720.6) | 249.92 (96.67, 1163.95) | 0.99 |
| **Baseline SCI (μmol/L)** | 0.38 (0.31, 0.56) | 0.45 (0.29, 1.26) | 0.30 |
| **Baseline Hepcidin (ng/ml)** | 134.39 (59.19, 243.70) | 60.15 (24.5, 249.05) | 0.32 |
| **OUTCOMES** | | | |
| **O2 need, n (%)** | 56 (60.2) | 14 (51.9) | 0.43 |
| **HFNC, n (%)** | 20 (21.5) | 4 (14.8) | 0.44 |
| **NIV, n (%)** | 18 (19.4) | 4 (14.8) | 0.59 |
| **CCU, n (%)** | 31 (33.3) | 9 (33.3) | - |
| **MV, n (%)** | 16 (17.2) | 5 (18.5) | 0.87 |
| **AKI, n (%)** | 8 (8.6) | 4 (14.8) | 0.34 |
| **AKI-KRT, n (%)** | 4 (4.3) | 3 (11.1) | 0.18 |
| **In-hospital Mortality, n (%)** | 17 (18.3) | 4 (14.8) | 0.67 |

Values are expressed as median (inter-quartile range) unless specified. Quantitative data are compared with Mann-Whitney test and Qualitative data are compared with chi-square test. RT-PCR negative group had more diabetics and also had higher N: L ratio

AKI- acute kidney injury, AKI-KRT- acute kidney injury requiring kidney replacement therapy, CCU- critical care unit, CRP- C-reactive protein, DM- Diabetes mellitus, HFNC- high-flow nasal canula, HT- hypertension, IL-6- interleukin-6, MV- mechanical ventilation, NIV- non-invasive ventilation, N:L ratio- neutrophil-to-lymphocyte ratio, RT-PCR- real-time polymerase chain reaction, SCI- serum catalytic iron, SI- serum iron, TIBC- total iron binding capacity, TSAT- transferrin saturation.

**Table E4: Coefficients of variation and lower limits of detection of the assays used in the study.**

| **Parameter** | **COV** | **Lower limit** |
| --- | --- | --- |
| **Catalytic Iron** | 5.4% | 0.03 µmol/l |
| **Ferritin** | 8% | 0.5 ng/ml |
| **Hepcidin** | 8.37 | 0.55 ng/ml |

COV: coefficient of variation
